# A Fluctuation-Driven Mechanism for Slow Decision Processes in Reverberant Networks

**DOI:** 10.1371/journal.pone.0002534

**Published:** 2008-07-02

**Authors:** Daniel Martí, Gustavo Deco, Maurizio Mattia, Guido Gigante, Paolo Del Giudice

**Affiliations:** 1 Computational Neuroscience Unit, Universitat Pompeu Fabra, Barcelona, Spain; 2 Institució Catalana d'Estudis Avançats (ICREA), Barcelona, Spain; 3 Department of Technologies and Health, Istituto Superiore di Sanità, Roma, Italy; 4 INFN, Sezione di Roma I, Roma, Italy; University of Cambridge, United Kingdom

## Abstract

The spike activity of cells in some cortical areas has been found to be correlated with reaction times and behavioral responses during two-choice decision tasks. These experimental findings have motivated the study of biologically plausible winner-take-all network models, in which strong recurrent excitation and feedback inhibition allow the network to form a categorical choice upon stimulation. Choice formation corresponds in these models to the transition from the spontaneous state of the network to a state where neurons selective for one of the choices fire at a high rate and inhibit the activity of the other neurons. This transition has been traditionally induced by an increase in the external input that destabilizes the spontaneous state of the network and forces its relaxation to a decision state. Here we explore a different mechanism by which the system can undergo such transitions while keeping the spontaneous state stable, based on an escape induced by finite-size noise from the spontaneous state. This decision mechanism naturally arises for low stimulus strengths and leads to exponentially distributed decision times when the amount of noise in the system is small. Furthermore, we show using numerical simulations that mean decision times follow in this regime an exponential dependence on the amplitude of noise. The escape mechanism provides thus a dynamical basis for the wide range and variability of decision times observed experimentally.

## Introduction

Over the last decade several experimental groups have identified neurons in association areas that participate in the decision making process. Electrophysiological recordings in the lateral intraparietal (LIP) area of macaque monkeys during random dot motion discrimination tasks have revealed that the activity of LIP neurons is correlated to the subject's choice and reaction time [1], [2] and is causally related to the decision formation [3]. When averaged over trials, LIP neurons show ramping activity with a slope modulated by the motion strength of the stimulus. These findings suggest LIP cells accumulate the sensory evidences needed to perform a perceptual decision (see [4], [5] for reviews)

A biologically-inspired cortical model that accounts for the observed decision-related neural activity of LIP was first proposed by Wang and colleagues [6], [7], [8]. The cortical model, based on the attractor paradigm [9], consists of a recurrent network of integrate-and-fire neurons with synaptic currents mediated by AMPA, NMDA and GABA receptors. Two subpopulations of strongly connected excitatory neurons encode the two possible choices in the decision task, and compete with each other for higher activity through feedback inhibition. Sensory moment-by-moment evidences, like those provided by MT cells that are selective to either of the two target directions in a random dot task [10], are modeled with specific external inputs to the competing populations. The activation of these inputs forces the network to change its state from a spontaneous activity state, in which both subpopulations show low firing activity, to an activated state, in which one of the subpopulations fires at a significantly higher rate than the other. The outcome of the decision is the choice associated with the winner population. The presence of noise in the system makes network decisions random.

The attractor model by Wang et al. provides a plausible explanation for the slowness of the decision mechanism, characterized by reaction times of the order of hundreds of milliseconds. Long reaction times arise in this model as a result of the attractor configuration of the system and the relatively large time constants of the NMDA receptor-mediated currents. The network, initially in the spontaneous state, is driven to a competition regime by an increase of the external input (that is, upon stimulus presentation) that destabilizes the initial state. The decision process can then be seen as the *relaxation* from an unstable stationary state [11] towards either of the two stable decision states. When the system is completely symmetric, i.e., when there is no bias in the external inputs that favors one choice over the other, this destabilization occurs because the system undergoes a pitchfork bifurcation for sufficiently high inputs [12]. The time spent by the system to evolve from the initial state to either of the two decision states is determined by the actual stochastic trajectory of the system in the phase space. In particular, the transition time increases significantly when the system wanders in the vicinity of the saddle that appears when the spontaneous state becomes unstable [7]. The transition can be further slowed down by setting the external input slightly above the bifurcation value [6], [7]. This tuning can be exploited to obtain realistic decision times.

In this work we explore an alternative mechanism for slow decision. Unlike the regime studied in [6], [7], here we focus on those cases where the stimulus does not destabilize the spontaneous state, but rather increases the probability for a noise-driven transition between the spontaneous state to one of the decision states. Due to the presence of finite-size noise in the system there is a nonzero probability that this transition occurs and hence a finite mean transition rate between the spontaneous and the decision states. We show that the proposed fluctuation-driven scenario for decision-making entails distinctive implications for the statistical distribution of the decision times. In particular we show, using numerical simulations, that mean decision times tend to the Van't Hoff-Arrhenius exponential dependence on the amplitude of noise [13], [14] in the limit of infinitely large networks. As a consequence, in this limit, mean decision times increase exponentially with the size of the network. It is also shown that, in the regime studied, the decision events become Poissonian in the limit of vanishing noise, leading to an exponential distribution of decision times. For small noise a decrease in the mean input to the network leads to an increase of the positive skewness of decision-time distributions. These results suggest that noise-driven decision models provide an alternative dynamical mechanism for the variability and wide range of decision times observed, which span from a few hundreds milliseconds to more than one second [15], [2].

## Results

### Decision making network

We use the decision making model introduced by Wang [6], based on a fully connected recurrent network of integrate-and-fire neurons and synaptic currents mediated by AMPA, NMDA, and GABA receptors [16] (see Materials and Methods for details). To assess the generality of the noise-driven mechanism, we also use a network which differs from the original in that the connectivity is sparse and synapses are instantaneous [17].

**Figure 1:**
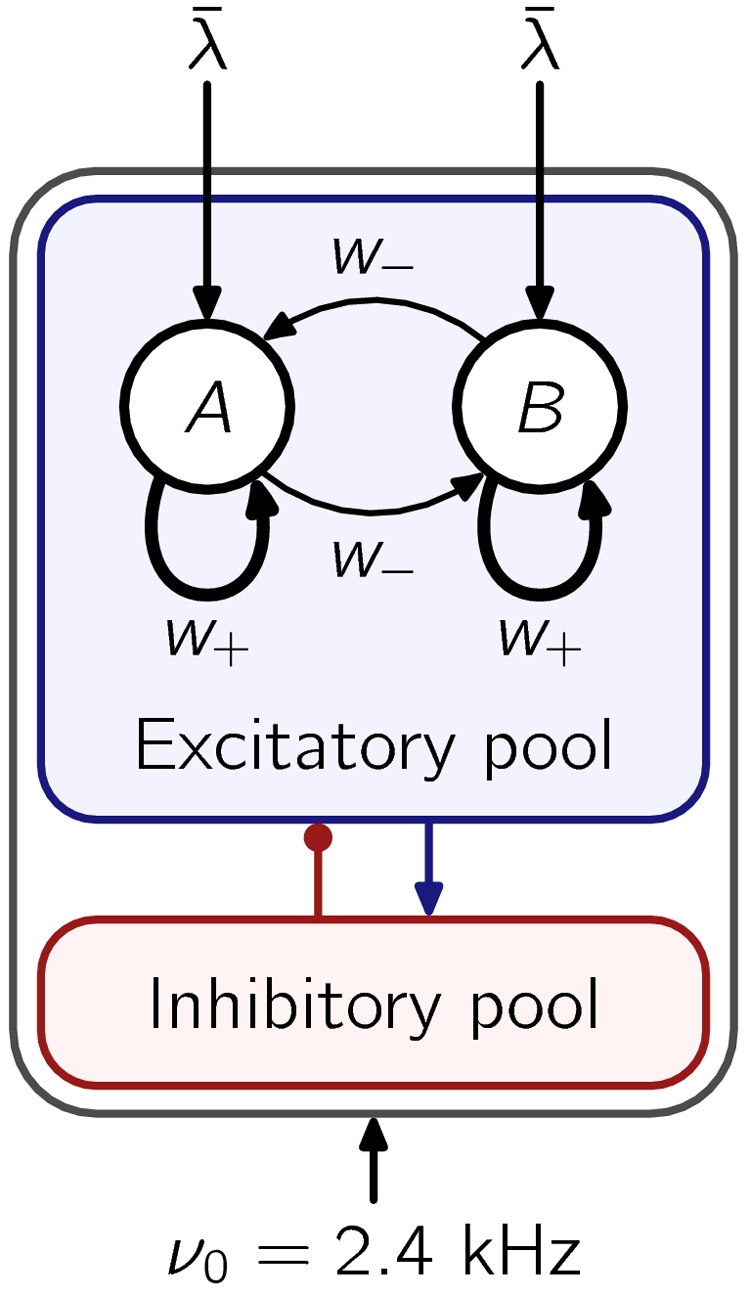
Architecture of the decision making network [6]. Two populations *A* and *B* of excitatory neurons encode the two possible choices in the decision process. They are endowed with strong recurrent connections (parametrized by *w*_+_), and inhibit each other through shared feedback from the inhibitory population. When recurrent connections are strong enough the network operates as a winner-take-all. Stimulation is modeled as an increase *λ̅*, with respect to the background external input *ν*_0_, in the rate of the Poisson spikes arriving to selective cells. See text for more details.

The network is structured in a set of different neural populations (see Figure 1). All neurons in the same population share the same statistical properties of the afferent currents and the connections. In the simplest model proposed by Wang, the network contains two subpopulations of excitatory neurons that encode the two possible choices to make, say *A* or *B*. These two *selective* populations (also labeled *A* and *B* according to the choice they encode) are connected to an inhibitory population, which is in turn connected to both neural groups. As a result of this shared inhibitory feedback, the two populations *A* and *B* compete with each other in a winner-take-all fashion when the external input is sufficiently high; the network eventually settles into a state where the activity of either one of the populations exceeds significantly the activity of the other. The choice made by the network is then said to be *A* if the activity of neurons in *A* is considerably higher than that of cells in *B*, and vice versa (see Network simulations for details).

The synaptic structure of the network is set according to the average inter-population synaptic efficacies that would result from a Hebbian plasticity mechanism [16]. Because neurons within a selective population tend to fire in a correlated way, connections between them are stronger than the baseline connection strength. We parametrize this relative potentiation by a factor *w*_+_>1. Analogously, connections between cells belonging to different selective populations are weaker than the baseline, because of the anticorrelation of pre- and postsynaptic firing. Connections from non-selective cells (that is, not belonging to *A* nor *B*) are also weakened by a factor *w*_−_>1. All other excitatory connections have relative strength *w* = 1. The baseline connection strength is given by the set of values for the recurrent excitatory conductances (*g*_AMPA,rec_ and *g*_NMDA_, in Materials and Methods) that allow the network to sustain spontaneous activity at physiological rates [17]. It is assumed that the spontaneous activity is not affected by synaptic modifications. This implies that, at the network level, the effect of synaptic potentiation must be compensated by synaptic depression, and hence that *w*_−_ must depend on *w*_+_ (see Materials and Methods).

Every cell in the network receives, apart from the recurrent currents, external currents which account for unspecific and uncorrelated activity of neurons outside the network. The activity of these external areas is modeled with independent Poisson spike trains of rate *ν*_0_ = 2.4 kHz. In addition to the background input, neurons from selective populations receive specific external input that accounts for information about stimuli (see Figure 1). This selective input is modeled with an increase in the rate of the incoming Poisson train from the background activity level *ν*_0_ to *ν*_0_+*λ̅*. We consider only identical inputs for the two selective populations, as the emphasis is on elucidating the differences between signal drive, relaxation decision dynamics and the noise-driven one. Those differences are expected to characterize the alternative decision dynamics also in the presence of bias in the input stimuli. In this configuration, the network chooses one or the other option with equal probability.

### Mean field analysis

In order to identify the attractors accessible to the network and to study their stability as a function of the parameters of the model, we used the mean field approximation derived in [16] (see s for a summary). The approximation allows to reduce the number of dynamical variables to the number of neural populations, and so it drastically reduces the computational cost associated with a scan in parameter space. The reduction in [18], [17], [16] provides the average firing rates of the different neuronal populations when: i) the number of neurons is infinitely large, ii) the unitary postsynaptic potentials elicited by presynaptic spikes are infinitesimally small iii) neurons from the same population share the same statistics of the input. We use this approximation to delimit the region of parameter space (*λ̅*, *w*_+_) where the network shows tristability among the spontaneous and the two decision states. We will later confirm with numerical simulations that the network of spiking neurons also shows tristability in approximately the same region.

As described in [7], there are essentially three qualitatively different network states, whose existence and stability depends on the parameter configuration. One is the symmetric state, characterized by the equal firing activities of the two selective populations. For low values of the self-excitation *w*_+_ and the external input *λ̅*, the firing activity of all excitatory neurons is around a few Hz; this corresponds to the network state we associate with the spontaneous activity in the cortex. The other two possible network states are the asymmetric (or decision) states, in which one selective population, either *A* or *B*, shows considerably higher activity than the other. These are the network states associated with the two categorical choices. Since the system is completely symmetric with respect to the transformation 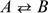, the decision states always appear and disappear in pairs as we vary the parameters. We will denote the coexistence of *A* and *B* with *C* (for competition).

**Figure 2:**
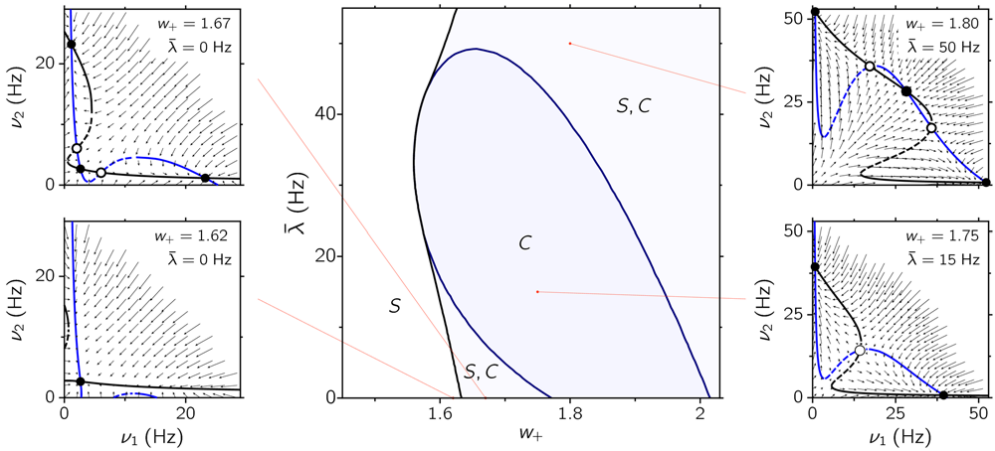
Central panel: Phase diagram of the system as given by the mean field approximation. In each region of the diagram, the presence of the different stable states is indicated by initials *S* (symmetric state) and *C* (competition, where both asymmetric states are present). In regions labeled with *S*,*C* there is tristability, all three states being simultaneously stable. Boundaries between regions correspond to bifurcation points at which either the symmetric state or the asymmetric states disappear (blue and black thick curves, respectively). Lateral panels show the fixed points, the flows, and the nullclines of the effective 2-dimensional reduction of the system (see Materials and Methods), for different representative points in the phase space. Filled and empty circles denote the stable and unstable fixed points of the reduced system. Black and blue curves are the nullclines *ν* ˙_2_ = 0 = 0 (horizontal flow) and *ν* ˙_1_ = 0 (vertical flow), respectively.

Figure 2 shows the regions where the different states are found, in the space of the specific input *λ̅* and the self-excitation *w*_+_. The existence and stability of every state was determined with the mean field approximation. The diagram shown is practically the same as the equivalent figure in [7], obtained with a further reduction of the mean field approximation we use. Note that there are no decision states when recurrent excitation is too low, no matter how strong the input is (see the *S* strip on the left of the phase diagram, and the phase portrait at lower left). The network lacks in this case the minimal degree of structure to sustain decision states. Figure 2 also shows that the minimal amount of recurrent excitation needed to have decision states depends non-monotonically on the input, as a consequence of the greater recruitment of shared inhibitory feedback for higher input strengths [16]. Importantly, there are regions of tristability (labeled *S*,*C* in the phase diagram), where the two asymmetric states coexist with the symmetric state. Note also that, although we distinguish two different (unconnected) regions of tristability in the phase diagram, they actually are portions of a connected region, as one would see if negative values for *λ̅* were included in the phase diagram. The stable symmetric states found at high enough *λ̅* and *w*_+_ (rightmost *S*,*C* region in the phase diagram; see also the upper right figure *w*_+_ = 1.80, *λ̅* = 50 Hz in the lateral panels) are characterized by firing rates considerably higher (≳20 Hz) than those associated with the spontaneous activity measured in the cortex. For this reason, we exclude this region from our analysis and concentrate on the *S*,*C* regime found between *w*_+_ = 1.6 and *w*_+_ = 1.8, for *λ̅*<20 Hz (lower center part of the phase diagram).

**Figure 3:**
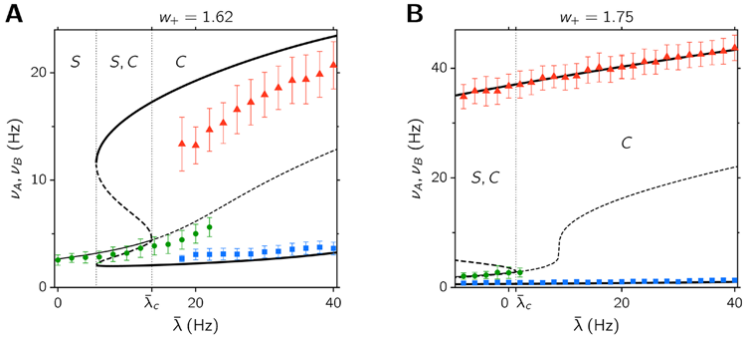
Dependence of the network activity on overall external input, as obtained from the mean field approximation (black solid curves) and from the network simulations (symbols), for *w*_+_ = 1.62 (A) and *w*_+_ = 1.75 (B). Thin solid curve: firing rates of both populations in a stable symmetric state (both populations at equal rates); thick solid curves: _ring rates of the two selective populations in an asymmetric state (one population at high rate, the other at low rate). Thick dashed lines show the position of the unstable fixed point. Dotted vertical lines indicate the boundaries of the three different regimes present in the system, as predicted by the mean field approximation. At *λ̅* = *λ̅* *_c_* the spontaneous state loses its stability. Error bars indicate the sample standard deviation of the firing rates.

The average firing rates of the symmetric and asymmetric states as a function of the selective input *λ̅* are shown in Figure 3, for two different values of the *w*_+_ lying in the region *S*,*C* considered. Solid curves in the figure are calculated from the mean field approximation, while data points are obtained from the network simulations. The discrepancies between simulations and mean field approximation are significant close to the bifurcation values, where fluctuations around the mean-field prediction are expected to be greater. Yet, both the mean field description and the simulations show that the network is able to sustain three different states for a given range of parameters *λ̅* and *w*_+_.

### Finite-size noise

In this model, network states are not really stable because of finite-size effects. Besides incoherent fluctuations (due to, e.g., quenched randomness in the neurons' connectivity and/or to external input), which are properly taken into account by the mean field approach through the variance of the input [17], corrections arise because for finite *N* the population spiking activity fluctuates around the infinite-*N*, mean field value.

These fluctuations induce transitions between the different network states and affect the collective dynamics of the network (see, e.g., [19]). We will use network simulations to capture the effect of finite-size noise on the stability of the network states. Although it is possible to incorporate finite-size effects in a mean field treatment [19], [20], [21], [22], [23] the description becomes too cumbersome when applied to complex architectures involving more than two populations recurrently interconnected, like those used in decision making networks (see [24] for an example using feed-forward architectures). The amplitude of finite-size effects can be controlled by using different network sizes and scaling proportionally the recurrent conductances, in such a way as to keep the average input current constant. In the simulations of the sparse network both the mean and the variance of the input current are kept constant as *N* varies.

### Network simulations

Once the ranges of parameters *λ̅* and *w*_+_ for which the network shows tristability were found, we studied the statistical properties of transition times and their dependence on the network parameters. To this end we simulated, given some fixed values for the parameters (*λ̅*, *w*_+_, *N*), 4000 trials with different random seeds, which determined the initial values for the membrane potentials and the synaptic gate variables, as well as the random realization of the external currents. With the first two parameters we controlled the regime of operation of the network (i.e., tristable or not), as well as the distance to the boundaries of the tristability range (*S*,*C*). By using different network sizes we modulated the amount of noise in the system.

To make the analysis simpler, and to mimic experimental conditions, we kept the value *w*_+_ of recurrent connectivity fixed and varied only the external input *λ̅*. The selected value of *w*_+_ was such that the spontaneous state was stable when *λ̅* = 0 and it was high enough to provide acceptable signal-to-noise ratios, the signal being the difference between the rate of the winning population and the rate of the spontaneous state, and the noise the amplitude of the rate fluctuations in the winning population. The value *w*_+_ = 1.75 fulfilled these two requirements. While keeping *w*_+_ fixed, we used *λ̅* as a control parameter that allowed us to drive the system from the tristable regime (*S*,*C*) to the competition regime (*C*) as well as to control the distance to the bifurcation point.

**Figure 4:**
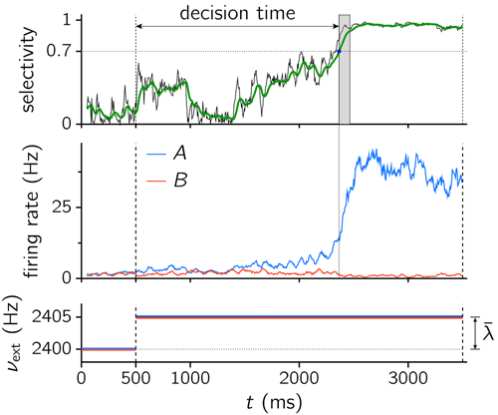
Evolution of the selectivity index (top), the average activity of populations *A* and *B* (middle), and stimulation applied (bottom), along a single trial. The green line in the top panel is the low-pass filtered selectivity with *τ* = 50 ms. From 0 to 500 ms no stimulation is applied (*λ̅* = 0). From 500 ms to the end of the trial, *λ̅* is set to a constant value different from zero (*ν*_ext_ = *ν*_0_+*λ̅* for selective cells). The *decision time* (DT) was the time elapsed between stimulus onset and the time at which the low-pass filtered selectivity index crossed the threshold 0.7 and stayed above it for at least 100 ms. The shaded area shows the time window within which the signal (green line) is required to be greater than the threshold. *N* = 2000, *w*_+_ = 1.75, *λ̅* = 5 Hz.

Every simulated trial consisted of two stages. During the first (pre-stimulus) stage, spanning from 0 to 500 ms, every neuron in the network received only the baseline background input. The network remained in the spontaneous state at that stage. After this period, neurons in both selective populations received an additional signal of magnitude *λ̅* (see lower panel in Figure 4 for a representation of the protocol used and the stimulation applied). This increase in input strength may either destabilize completely the spontaneous state or facilitate noise-induced transitions to the decision states.

The occurrence of a transition in the simulated trial was determined with the selectivity index defined as *X* = |*ν_A_*−*ν_B_*|/(*ν_A_*+*ν_B_*). This variable provides a measure of the asymmetry between the two rates and allows to describe with a single variable the transition from the spontaneous state (*X*≳0) to a decision state (*X*≲1, see top panel in Fig. 4). The selectivity index *X* can thus be thought of as the ‘decision variable’, or weight of evidence supporting one alternative over the other in the decision problem [25,25]. Furthermore, to take into account occasional high fluctuations transiently bringing the selectivity index *X* above threshold, we applied a first-order low-pass filter with *τ* = 50 ms and considered that a decision was properly formed if the filtered signal crossed the threshold *X*_thr_ = 0.7, and remained above it for at least 100 ms. We name decision time (DT) the time elapsed between stimulation onset and threshold crossing. The criterion used differs from the ‘hard threshold’ methodology used in [6], [7], but it leads to qualitatively similar results and it has the advantage of avoiding the use of a particular level of activity as threshold.

**Figure 5:**
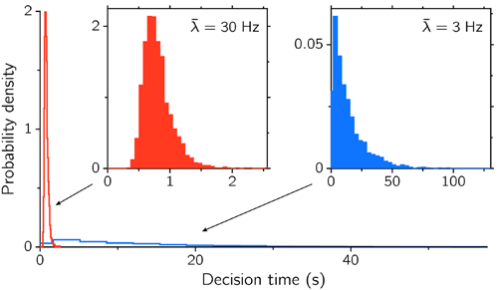
Distributions of decision times for a regime with spontaneous stable state (blue, *λ̅* = 3 Hz) and without spontaneous stable state (red, *λ̅* = 30 Hz), from a sample of 4000 trials each. The two insets show the distributions separately (note the different scales). *N* = 4500, *w*_+_ = 1.75.

According to the bifurcation diagram in Fig. 3B, given *w*_+_ = 1.75 the spontaneous state is stable for values of *λ̅* below the value *λ̅* *_c_* = 2 Hz, approximately. Figure 5 presents the distribution of DTs for two values of *λ̅*: one below (blue) and one above (red) the critical value *λ̅* *_c_*. For low input intensities (*λ̅*<*λ̅* *_c_*) transitions between network states are fluctuation-driven, and the distribution of transition times is very skewed right, close to an exponential or a gamma with very low shape parameter. In contrast, high enough input intensities lead to transition times that are significantly shorter, more narrowly distributed, and less right skewed as a consequence of the dominant deterministic mechanism underlying the transition [11].

**Figure 6:**
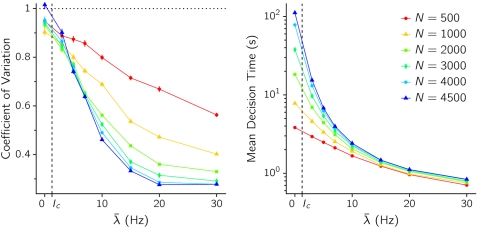
Coefficient of Variation (A) and mean value (B) of decision times versus the external input intensity *λ̅*, for different sizes of the network, as indicated in the key. *w*_+_ = 1.75.

The transition from a fluctuation-driven to a relaxation regime is more abrupt the lower is the presence of noise in the system. This is shown in both panels in Figure 6, where the mean value and coefficient of variation (CV) of decision times obtained from a simulated sample are represented as a function of the control parameter *λ̅* for different levels of noise. Mean decision times grow as the external input is reduced, regardless of the regime in which the network operates. Decision times are however much more sensitive to the value of *λ̅* in the fluctuation-driven regime than in the relaxation regime.

Second order statistics of decision times also show distinctive properties depending on the regime. The variability of decision times around the mean is measured with the coefficient of variation, CV = σ_CV_/〈CV〉, and is plotted in Fig. 6A. The CV of DTs tends for sufficiently large *N* (small noise) to the value 1 as *λ̅* is decreased below the bifurcation value. This asymptotic value, together with the histogram in Fig. 5 (blue), suggest that in this regime and in the limit of vanishing noise decisions are essentially Poisson processes, with exponentially distributed decision times. This Poissonian character is gradually lost as the external input increases and the deterministic component of the dynamics takes over the stochastic one, leading to more peaked, gamma-like DT distributions and hence to lower CV values. From Fig. 6A it is also seen that for *λ̅*<*λ̅* *_c_* the value of CV of DT is essentially insensitive to the amount of noise (while the mean value of DT strongly depends on *N* in the same region), consistently with the picture of an approximate Poisson statistics for the noise-driven decision process. For *λ̅*>*λ̅* *_c_* the converse is observed, the strong dependence of CV on *λ̅* being due to the fact that for increasing noise (decreasing *N*) the representative point in the (*ν_A_*, *ν_B_*) plane drops off the symmetric ridge down from the unstable spontaneous state at more widely distributed times.

**Figure 7:**
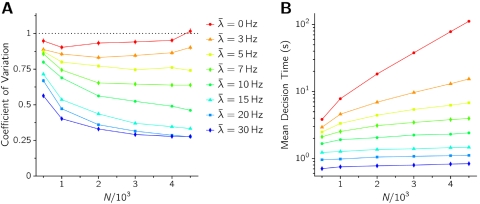
Coefficient of Variation (A) and mean value (B) of decision times versus the size of the network, for different external input intensities, as indicated in the key.

According to the theory of stochastic processes, the average escape time from a metastable state in a unidimensional system depends exponentially on the inverse of the variance *σ* ^2^ of the fluctuations (Van't Hoff-Arrhenius law): 〈*T*〉∼exp(Δ*U*/*σ* ^2^), where Δ*U* is height of the potential barrier the system has to jump over to escape from the basin of attraction of the initial state. For multidimensional systems it may even be impossible to define a potential function, but the general dependence on *σ* ^2^ is still of the type ∼exp(*K*/*σ* ^2^) [13], [14]. In any case, since *σ* ^2^ scales as 1/*N*, decision times grow exponentially with the size of the network. As Fig. 7B shows, the mean DT does indeed grow exponentially with *N* for *λ̅*<*λ̅* *_c_*, consistent with the theory of noise-driven escape processes. Furthermore, the CV tends to one as *N*→∞ for *λ̅*<*λ̅* *_c_*, while it slowly decreases with *N* when *λ̅*>*λ̅* *_c_* (Figure 7A). In the thermodynamic limit *N*→∞ the CV would decay to 0 whenever *λ̅* is high enough to destabilize the spontaneous state, as the transition would consist in this case on a deterministic relaxation from an unstable to a stable state.

**Figure 8:**
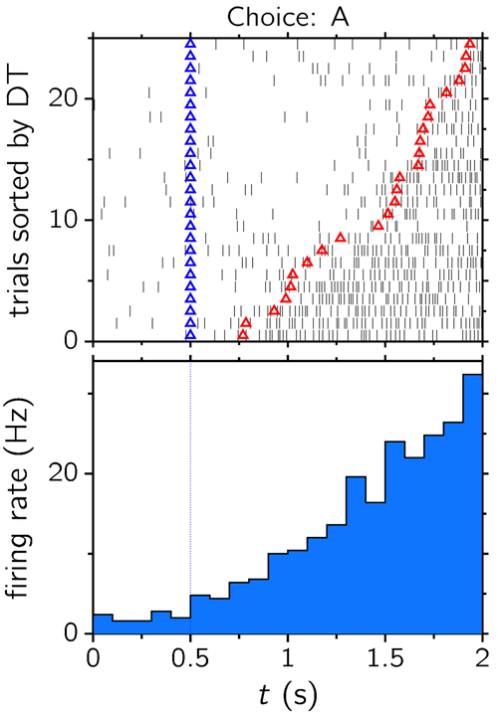
Upper row: rasters of a single cell in population *A*, across 20 different trials and sorted by decision time. Bottom row: trial-averaged firing rate. In all these 20 trials the network chose option *A* (in physiological terms, the target choice is in the response field (RF) of the recorded cell). Blue triangles: stimulus onset. Red triangles: decision time, determined following the criterion shown in Figure 4. *λ̅* = 5 Hz, *w*_+_ = 1.75, *N* = 500.

The decision dynamics unfolded in this regime are compatible with the ramping-like activity observed in LIP when neuronal activity is averaged over trials [1], [2]. In the noise-driven regime, single trial activity exhibits a rather sharp transition between the spontaneous and an activated state. Such abrupt transitions are illustrated in Figure 8, which shows simulated single-cell raster activity from different trials (top) and the corresponding trial-averaged activity (bottom; see also Fig. 4 for the population averaged activity). Even if transitions are thought of as sharp, random jumps between two stereotyped levels of activity, smooth ramping activities are obtained when averaging over trials [26], [27], [28], [29]; namely, if *r*(*t*) = Θ(*t*−*T*), where *r*(*t*) is the cell activity, Θ is the Heaviside function, and *T* is the decision time for a given trial (a random variable drawn from some probability density function), the average over trials gives rise to the cumulative density function of the decision times, 〈*r*(*t*)〉 = Prob(T<*t*). The fact that realistic cumulative density functions are smooth, monotonically increasing functions would explain in this case the ramping activity observed in trial-averaged activities (see also Discussion).

We show in the next section that the picture emerging from Figs. 6 and 7 is recovered in simulating sparse networks of simpler synaptic and neural elements, where larger *N* intervals have been explored.

### Sparse network

In this Section we briefly discuss the results of an analysis similar to the one performed in the previous Sections, but carried out in the context of a simpler network model. Specifically, the network is again composed of four populations of leaky integrate-and-fire neurons, with the same architecture as in the previous Sections, (for *N* neurons, 12% of *N* belong to each of the selective, *A* and *B* populations, 20% of *N* are inhibitory neurons, and 56% of *N* are background, non-selective excitatory neurons) with the following differences:

1. synaptic transmission is instantaneous: the dynamics of AMPA, NMDA and GABA receptors are totally ignored.
2. the connectivity is sparse: every neuron in the network receives spikes from a fixed number of presynaptic neurons, randomly chosen at the beginning of the simulation. Hence, no topology is imposed on the network structure. This random choice of synaptic connectivity provides a source of ‘quenched’ noise, such that simulations run for the same set of parameters and the same stimulation protocol embody different realizations of the statistical distribution of synaptic contacts.
3. spikes are propagated to their postsynaptic targets with a delay δ. The values of δ are drawn from an exponential distribution with mean value 〈δ〉 = 11.3 ms for spikes generated by excitatory neurons, and 〈δ〉 = 1.2 ms for inhibitory spikes. A distribution of spike transmission delays is a physiologically plausible feature to incorporate, and contributes to make the states of asynchronous activity of the network more stable, tempering the propensity to ignite global oscillations [30]. We remark that the longest delays between excitatory neurons are much smaller than the characteristic time of NMDA conductances.

The values of the synaptic efficacies are chosen such that the unstructured network (*w*_+_ = *w*_−_ = 1) possesses a stable state of spontaneous activity with *ν_E_* = 3 Hz and *ν_I_* = 6 Hz for the excitatory and the inhibitory neurons, respectively. Along the lines of the previous Sections, the symmetry between the self-excitation and the cross-excitation in the populations *A* and *B* is broken by choosing *w*_+_>1 and *w*_−_<1 in such a way as to support three fixed points in the network (*S*,*C*).

The main purpose of this stage of analysis is to illustrate how the purely noise-driven mechanism envisaged is able per se to account for slow decision processes in the simplest network model, implicitly checking whether the characteristic times of the synaptic transmission, included as realistic features in the Brunel-Wang model adopted in the previous Sections, are essential in allowing the network to exhibit such a wide range of DTs. We will show that, indeed, mean DTs obtained from the simplified network studied in this Section also extend to very high values compared to all synaptic times, including those associated with NMDA.

**Figure 9:**
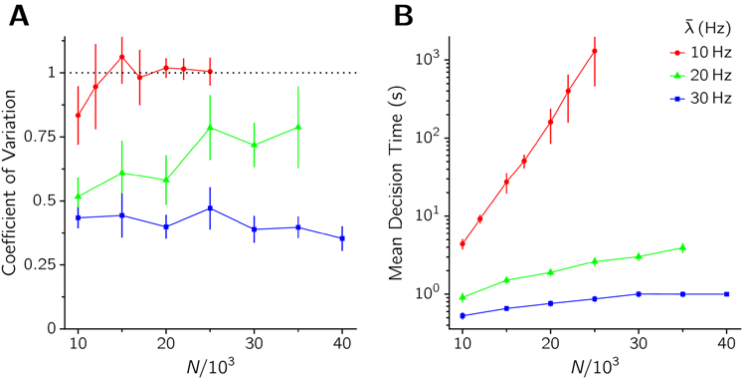
A: Coefficient of variation CV of Decision times, DT vs size of the sparse network *N*. B: Mean DTs vs *N*.

The stimulation protocol and the estimate of DT are the same as in the previous Sections. For a given set of network parameters, statistics on the DT is accumulated over 200 simulations. Consistently with theoretical expectation, the long tail of the distribution of DTs is well fitted by an exponential. Results are summarized in Fig. 9 in terms of the mean decision times and their coefficient of variation with respect to the network size *N*, for three values of *λ̅* = 10 Hz, *λ̅* = 20 Hz, and *λ̅* = 30 Hz (*w*_+_ = 1.41).

According to the mean field approximation the spontaneous state is stable for the first two values of *λ̅* and unstable for the third. From Fig. 9B it is seen that when the stimulus intensity *λ̅* is such as to keep the spontaneous state stable, the mean DT is very close to be exponential in *N*, confirming the scenario of noise-driven transitions reported in the previous Section. For strong stimuli (see the case *λ̅* = 30 Hz in the figure) mean DTs show a very mild dependence on *N*, confirming the quasi-deterministic nature of the motion. Furthermore, as Fig. 9A shows, the CV of the noise-driven escape events (*λ̅* = 10 Hz and 20 Hz) tends to 1 for sufficiently small noise (large *N*) thereby signaling an asymptotic Poisson behavior, which is consistent with known theoretical results on the distribution of residence times in noisy bistable systems. For *λ̅* = 30 Hz CV stays approximately constant for the whole of the range explored for *N*, with a slight decrease for high *N*. Indeed, when the stimulus destabilizes the initial spontaneous state and the noise is very small, intuition suggests that the CV of DT should tend to zero (for a symmetric landscape in the phase space).

The simplification introduced in the present Section aims to show that in the mechanism we adopted, of a noise-driven escape from the spontaneous state as the dynamical underpinning of the decision process, very slow decision times can be easily obtained as a result of the interaction between the finite-size noise and the cooperative-competitive dynamics of the system.

## Discussion

Prominent features emerging from a variety of psychophysics experiments implying a choice between alternatives (either conscious or unconscious, as in binocular rivalry), are the very wide range of decision times and their variability [15], [31], [2], with distributions exhibiting long tails that can be approximated as exponential (see e.g. [32]). The present work wants to demonstrate that a stimulus-triggered and noise-driven mechanism operating in a very simple, prototypical neural architecture, can easily accommodate such features, without need of fine-tuning, and in a robust way with respect to the details of the neuron and synapse models. Given that we investigated only the case of unbiased stimuli, the phenomenology associated with the psychometric curves, usually adopted to quantify behavioral performances, is outside the scope of the present work. Predictions amenable to experimental check include those related to the shape (skewness) of the distribution of decision times for the relaxation and the noise-driven regimes for maximally ambiguous stimuli. This work contributes clear evidence, in a simple setting, that noise-driven transitions among stable, asynchronous collective network states constitute a viable and robust mechanism for spanning ‘behavioral times’ far beyond all the time scales of the neural and synaptic dynamics. This idea has been explored in the context of perceptual alternations in a recent paper by Moreno-Bote *et al.* [33], which proposes a broad class of attractor-based models that are consistent with salient properties of perceptual rivalry phenomena. Among these, the model accounts for the observed narrow distribution of dominance durations as well as for the associated mean value of the order of seconds, substantially higher than the intrinsic synaptic and neuronal time scales. The principle of noise-driven transitions has also been appealed to explain the neurophysiological and behavioral signatures of Weber's law, in the context of a two-forced choice vibrotactile task [34].

Attractor models describe the decision process as a stimulus-triggered transition from the spontaneous activity state and a decision state decision state associated with a given categorical choice. Noise is a constitutive ingredient of these models, and is responsible for the probabilistic outcome of the decision process. The effects of noise on the system, however, depend strongly on the network regime. Previous works on attractor models of decision have focused on the regime where the inputs were strong enough to destabilize the spontaneous activity state, forcing the network to relax to either of the decision states. In this relaxation regime noise does not induce the transition but it rather introduces randomness in the process, by making the system wander around the separatrix delimiting the basis of attraction associated with each choice. Once the system leaves this region, the deterministic component of the dynamics takes over and makes the system decay to the final attractor.

A different mechanism for decision emerges when the mean input is low and does not destabilize the spontaneous state. In this case there is tristability among the decision and the spontaneous states, and the dynamics of the transition are genuinely different from the relaxation mechanism. The existence of such multistable regime is a plausible assumption supported by the observation of delay activity during delay-response versions of the random dot discrimination task [1], [2]. It is also reasonable to assume that this multistability is not destroyed when the inputs are low enough, due to structural stability. Under these conditions, noise plays a primary role in the decision process by letting the system surmount the energy barriers and jump from the spontaneous state to either of the decision states.

Although the whole analysis has been confined to balanced inputs for the two competing neural populations, we stress that the conclusions drawn in this work also apply to situations with asymmetric inputs. When this is the case, one of the selective neural populations receives more input than the rest, and the basin of attraction of the corresponding attractor grows at the expense of the others. This change in the attractor landscape results in a greater probability of choosing the option favored by the inputs [7]. Regardless of these modifications in the attractor landscape, the spontaneous state will remain stable as long as the inputs (balanced or not) are low enough not to destabilize it.

The main prediction that follows from this work is the existence of two distinguishable decision behaviors depending on the mean input feeding the decision network. For low inputs, the dynamics governing the decision process are mainly noise-driven and characterized, in the limit of vanishing noise, by exponentially distributed decision times, with coefficients of variation close to 1, and mean values that can be substantially larger than neuronal and synaptic time scales. On the other hand, when the mean input is higher than a critical value, we expect decision times to be less variable and, on average, to decrease monotonically with the input. These predictions become more exact the smaller is the amplitude of the noise present in the system.

Several factors may contribute to the modulation of the overall afferents to LIP. The neuronal activity in LIP is affected not only by the motion information provided by the projections from MT, but also by the temporal structure of the task [1]. When the trial is short and the subject has to make a rapid decision, the neuronal activity in LIP evolves more rapidly than when trials are longer. Thus, the expectations of the subject about the duration of the trial influence the evolution of the neuronal activity in LIP. Also the behavioral value associated with each choice has been found to modulate the activity in LIP cells [35]. All these modulations are likely to be attributable to changes in the afferent activity to LIP. Interestingly, a recent analysis of the generic dynamical properties of winner-take-all networks shows that modulations in the input common to both populations can account for the speed-accuracy tradeoff observed in behavioral experiments [12]. From this analysis it follows that the pre-stimulus average activity in LIP should be higher when the subject has to respond more rapidly, and it should decrease when the subject has to respond more accurately. If the prediction is correct, the overall input to the decision making network could be manipulated by instructing subjects to respond before a given time deadline [36], [37]. Thus, a shortening of the time deadline would result in an increase of the overall input feeding both populations, and the network would operate in a relaxation regime. Similarly, long time deadlines allow for very accurate responses and presumably entail low common inputs to the decision network. If two different mechanisms for decision exist, one should observe that reaction time distributions tend to be more skewed in long duration trials, where the subject sacrifices speed for accuracy.

Furthermore, since the final response time is a sum of the decision time and some residual (transduction, transmission, etc.) latencies, we expect response time distributions to reflect only in part the time devoted to decision formation [15]. Although these residual latencies are relatively short compared to the decision times when the discrimination is difficult, they introduce an additional source of variability in the final response time, whose distribution necessarily reflects the indeterminacies of both decision and non-decision contributions. In this respect, exponentially distributed decision times cannot be ruled out on experimental grounds. In fact, it has been suggested that the long right tails observed in empirical response-time distributions may result from the contribution of some exponentially distributed random variable in the response time [15]. A simple description that explores this exponential contribution is the ex-Gaussian model [38], [15], [39]. In this model, response times are the sum of two independent random variables: one, exponentially distributed, represents the decision stage, while the other, normally distributed, represents the nondecision stage. The distribution of response times is given in this case by the convolution of an exponential and a normal distribution, which is an ex-Gaussian. This distribution turns out to fit surprisingly well behavioral data.

We have also showed that, for a noise-driven decision scenario, the widely distributed decision times can give rise to a ramping profile of the trial-averaged firing rate. Such profiles have been observed in experiments involving perceptual decisions making, and we suggest that sharp firing rate transitions sparsely occurring in time, as implied in the present work, might also contribute to the explanation of these observations. This is not meant to exclude ramping firing activity at the single trial level; indeed, published data would seem to provide partial support to both scenarios [40], [41]. Perceptual decisions leading to motor responses such as saccades would plausibly involve a multi-stage process, first accumulating perceptual evidence, to be later read out by downstream neurons. In a noise-driven decision scenario, ramping activities observed in peristimulus time histograms would be an artifact of averaging single trials characterized by sharp firing rate transitions for the first stage, and a genuine reflection of single trial features for the second stage.

In this work the noise source is explicitly identified as the finite-size fluctuation of the network spiking activity. As such, it does not affect the dynamics as an additional preset external random signal (as in several analysis previously proposed), but rather as a re-entrant effect of the network recurrent dynamics. While the effective number *N* of neurons involved in the various stages of a decision process is obviously unknown, and the predictions shown for the *N*-dependence of the decision times statistics cannot be directly checked, a qualitative hint might come from experiments in which different stimulation/performance conditions are thought to involve neural populations of different sizes in the same brain areas. For example, it has been suggested that the ‘oblique effect’, by which subjects discriminate better visual stimuli with horizontal and vertical rather than oblique orientations, may result from the overrepresentation of cardinal (horizontal/vertical) orientations in MT cells [42]. If a similar anisotropic representation of orientations is found in LIP, one could devise an experiment showing different distributions of reaction times depending on the orientation of the opposing targets in a random-dot direction discrimination task. For instance, a subject can be instructed to respond within different time intervals so that one can manipulate the speed-accuracy tradeoff [37]. The average reaction time in long duration trials should be longer for choices involving cardinal orientations than for choices in oblique orientations, by virtue of the different number of cells involved in their representation. The higher amount of noise associated with the representation of oblique orientations would also account for the ‘oblique effect’ itself, as larger noise amplitudes give rise to poorer performances.

## Materials and Methods

We use the network introduced by Brunel and Wang (2001), with the same parameters. For more details about the choice of the parameters, please refer to the original article and the references therein.

### Network

The network consists of *N_E_* excitatory neurons (80%) and *N_I_* inhibitory neurons (20%). Each neuron receives from the network *N_E_* excitatory synaptic contacts and *N_I_* inhibitory synaptic contacts; the network is thus fully connected. The whole set of neurons is partitioned into different populations, all neurons in a population sharing the same statistical properties of the afferent inputs. The set of all excitatory neurons is in turn structured in three different populations: two populations formed by neurons that encode one or the other choice, and a third population formed by the remaining excitatory neurons. The former two constitute the two disjoint *selective* populations, of *fN_E_* (*f* = 0.15) neurons each. The other (1-2*f*)*N_E_* excitatory neurons do not encode any information about the choices, and constitute the *non-selective* population. To simulate the background input from other brain regions, every neuron in the network receives 800 excitatory connections from external neurons, each of which fires according to an independent Poisson process with rate 3 Hz.

### Neurons

Neurons in the network are described by leaky integrate-and-fire (ir) neurons with resting potential *V_L_* = −70 mV, firing threshold *V*_thr_ = −50 mV, reset potential *V*_reset_ = −55 mV, and refractory period *τ_rp_* = 2 ms for excitatory cells and *τ_rp_* = 1 ms for inhibitory cells. The subthreshold dynamics of the membrane potential, *V*(*t*), of each ir neuron obeys:
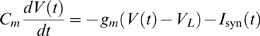
where *C_m_* is the membrane capacitance, with value 0.5nF for excitatory neurons and 0.2pF for inhibitory neurons; *g_m_* is the membrane conductance, and is set to 25nS for excitatory cells and to 20nS for inhibitory cells. The total synaptic current into the cell, *I*_syn_(*t*), is a sum of recurrent (coming from the local module) and external (background activity and stimuli) contributions, described in detail below.

### Synapses

The synaptic current includes glutamatergic excitatory components (mediated by AMPA and NMDA receptors) and inhibitory components (mediated by GABA). External excitatory contributions operate only through AMPA receptors (*I*_AMPA,ext_). Thus the total synaptic current is
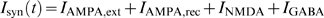
(1)where the different components are given by
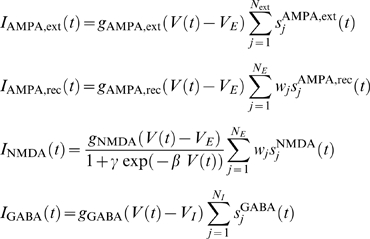



The reversal potentials are *V_E_* = 0 mV and *V_I_* = −70 mV. To keep the mean recurrent input constant as we vary the size *N* of the network, all recurrent conductances are rescaled by 1/*N*. The dimensionless parameters *w_j_* found in the excitatory recurrent currents introduce structure in the excitatory connections (see ‘Connectivity structure’, below). The values for the synaptic conductances for excitatory neurons are *g*_AMPA,ext_ = 2.08nS, *g*_AMPA,rec_ = 104nS/*N*, *g*_NMDA_ = 327nS/*N*, and *g*_GABA_ = 1250nS/*N*. For inhibitory neurons *g*_AMPA,ext_ = 1.62nS, *g*_AMPA,rec_ = 81nS/*N*, *g*_NMDA_ = 258nS/*N*, and *g*_GABA_ = 973nS/*N*. NMDA currents are voltage dependent and modulated by intracellular magnesium concentration [Mg^2+^] = 1.0 mM, with parameters *γ* = [Mg^2+^]/(3.57 mM), *β* = 0.062(mV)^−1^. The fraction of open AMPA (external and recurrent) channels, *s*_AMPA,*j*_ in neuron *j* follows the dynamics:
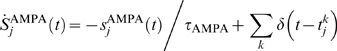
where *τ_AMPA_* = 2.0:*ms*, and the sum over *k* represents a sum over spikes emitted by presynaptic neuron *j* at time *t_k,j_*. In the case of external AMPA currents, the spikes are fired following a Poisson process with rate *ν*_ext_ = 2.4 kHz, except for the selective populations, which receive a Poisson spike train with rate *ν*_ext_ = 2.4 kHz+*λ̅*. The dynamics for the NMDA synaptic currents are described by
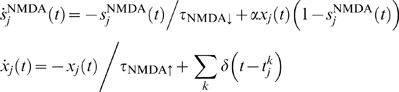
where characteristic rise and decay times are *τ*_NMDA↑_ = 2.0 ms and *τ*_NMDA↓_ = 100 ms, and *α* = 0.5(ms)^−1^. The GABA synaptic component obeys the equation
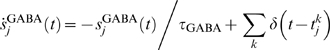
with *τ*_GABA_ = 5 ms.

For the sparse network we used instantaneous synaptic transmission:
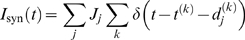
where *j* labels all presynaptic neurons, *J* is the synaptic efficacy, 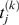 is the time of the *k*-th spike emitted by the *j*-th presynaptic neuron, and 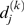 is the corresponding transmission delay.

### Connectivity structure

Connection weights between different populations determine the structure and function of the network. Weights are given by the parameters *w_j_* (see equations following eq. (1)), which denote the relative strength of the modified synapses with respect to the baseline, to which there corresponds the value 〈*w*〉 = 1. Note that only recurrent currents (*I*_AMPA,rec_, *I*_AMPA,ext_, and *I*_GABA_) contain weights, the precise, fixed values of which are assumed to be determined by some Hebbian learning mechanism, not simulated in this work. According to this mechanism connection weights *w_j_* are high when the activity of pre and postsynaptic neurons is correlated, low when it is anticorrelated, and unaltered (equal to 1) when it is uncorrelated. In a selective population, where neurons tend to be coactivated, connections are strengthened above the baseline. The connection weight *w_j_* inside a selective population is a measure of the recurrent self-excitation and is denoted by *w*_+_>1. Analogously, since the activity of the two selective populations is anticorrelated, the two populations are weakly connected, with a value denoted by *w*_−_<1. All other weights are set to the baseline value 1. To ensure that the average excitatory synaptic efficacy is not changed in the learning process, *w*_−_ must depend on *w*_+_ as 1−*f*(*w*_+_−1)/(1−*f*) [17].

### Simulations

We have used a 2nd order Runge-Kutta routine to integrate the system of coupled differential equations that describe the dynamics of all cells and synapses. The time step used was 0.02 ms. To calculate the firing rate of a population we divided the number of spikes emitted in a 50 ms window by the number of neurons in the population and by the window size. The time window was slided with a time step of 5 ms. Every trial was simulated until a decision was made. For a given parameter set, we estimated decision times from a block of 4000 trials. The sparse network is simulated using an event-driven, exact approach [43].

### Mean field approximation

We use the mean field approximation derived in [16], which yields a set of *n* nonlinear equations describing the average firing rate of the different populations in the network:
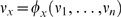
(2)where *x* = 1,…,*n* labels the different neural populations, and different *φ_x_* is a nonlinear function providing the output rate of a population *x* in terms of the inputs, which depend in turn on the rates of all the populations. The system of equations (2) expresses then the self-consistency condition that neurons in every population produce an output that is compatible with their inputs. To solve the system (2) we integrate numerically the set of differential equations
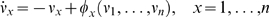
(3)which have the same fixed point solutions as equations (2). To find all the possible fixed-points that coexist for a given parameter setting, we integrated the equations (3) with different initial conditions. The explicit expressions for (2) can be found in [16].

### Nullclines and rate-flow diagrams

We apply the dimensional reduction presented in [44] to the mean field equations (2). The basic idea is to consider *l* of the *n* variables of the system as parameters; while keeping these *l* parameters fixed, we find the stationary points of the remaining *n*-*l* rate variables by using eqs. (3). That is, we allow the system to adapt to the stationary state induced by the *l* frozen variables. The nullclines shown in Figure 2 are calculated by quenching one of the rates associated with either of the two selective populations. For example, the nullcline *ν* ˙_2_ = 0 is obtained by taking *ν*_1_ = *ν̅* as a parameter of the system and calculating, for every value of this parameter, the solutions of
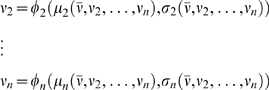
(4)


The values (*ν*_2_,…,*ν_n_*) that satisfy eqs. (4) are the fixed-points of the (*n*-1)-dimensional map defined by the equations. Note that for a given value of the parameter *ν̅* there may exist different fixed-points due to the non-linearity of the transfer functions φ. The nullcline *ν* ˙_2_ = 0 is then obtained by plotting the values of that one gets after solving (4), against the value of *ν*_1_ = *ν̅*, for all the values of *ν̅* in the range considered. The nullcline *ν* ˙_1_ = 0 is obtained in a completely analogous way, taking as quenched variable.

The rate-flow diagrams were plot following the same principle. We covered a part of the ν_1_–ν_2_ plane with a grid. At each point (*ν̅*_1_,*ν̅*_2_) of this grid we solved the (*n*-2)-dimensional fixed-point equation:
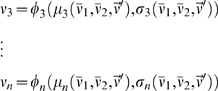
(5)where 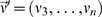 is the rate vector formed by the dynamical (not quenched) variables. For fixed rates *ν*_1_ and *ν*_2_, therefore, the solutions of (5) are the stationary points of the remaining populations induced by the rates quenched at *ν*_1_ = *ν̅*_1_ and *ν*_2_ = *ν̅*_2_ and by the full feedback among all the other populations. The solution depends on *ν̅*_1_ and *ν̅*_2_, i.e., 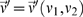. The currents afferent to neurons in populations 1 and 2 tend to drive them to new rates *ν*_1,out_ and *ν*_2,out_ that are in general different from the quenched values *ν̅*_1_ and *ν̅*_2_:
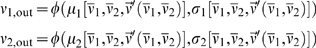
The rate-flow diagram was obtained by drawing at each point of the grid an arrow from the point (*ν*_1_,*ν*_2_) to (*ν*_1,out_,*ν*_2,out_). For clarity we represented every arrow with a length given by log(1+*m*/2), where *m* is the original length of the arrow, and excluded arrows whose modules were larger than 8.0.

### Coefficient of variation

The CV was estimated by the ratio of the sample mean, 
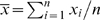
 to the sample standard deviation, 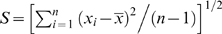, of the decision times obtained from the *n* simulated trials. To calculate the error associated with the estimated CV we used the independence of the sample mean and the sample s.d., and the fact that the variances of the estimators *x̅* and *S* ^2^ are, respectively, 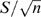 and [*μˆ*_4_−(*n*−3)/(*n*−1)*S* ^4^]/*n*, where 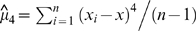.
